# Investigating the circulating sphingolipidome response to a single high-intensity interval training session within healthy females and males in their twenties (SphingoHIIT): Protocol for a randomised controlled trial

**DOI:** 10.12688/f1000research.128978.3

**Published:** 2023-08-18

**Authors:** Justin Carrard, Thomas Angst, Nadia Weber, Joëlle Bienvenue, Denis Infanger, Lukas Streese, Timo Hinrichs, Ilaria Croci, Christian Schmied, Hector Gallart-Ayala, Christoph Höchsmann, Karsten Koehler, Henner Hanssen, Julijana Ivanisevic, Arno Schmidt-Trucksäss

**Affiliations:** 1 Division of Sport and Exercise Medicine, Department of Sport, Exercise and Health, University of Basel, Basel, 4052, Switzerland; 2 Cardiac Exercise Research Group, Department of Circulation and Medical Imaging, Norwegian University of Science and Technology, Trondheim, Norway; 3 Sports Cardiology Section, Department of Cardiology, University Heart Center Zurich,, University Hospital Zurich, Zurich, 8091, Switzerland; 4 Metabolomics Platform, Faculty of Biology and Medicine, University of Lausanne, Lausanne, 1005, Switzerland; 5 Department of Sport and Health Sciences, Technical University of Munich, Munich, Germany

**Keywords:** Cardiometabolic health, cardiovascular heatlh, exercise, physical activity, sphingolipids, ceramides, exercise medicine

## Abstract

**Introduction: **Growing scientific evidence indicates that sphingolipids predict cardiometabolic risk, independently of and beyond traditional biomarkers such as low-density lipoprotein cholesterol. To date, it remains largely unknown if and how exercise, a simple, low-cost, and patient-empowering modality to optimise cardiometabolic health, influences sphingolipid levels. The SphingoHIIT study aims to assess the response of circulating sphingolipid species to a single session of high-intensity interval training (HIIT).

**Methods: **This single-centre randomised controlled trial (RCT) will last 11 days per participant and aim to include 32 young and healthy individuals aged 20-29 (50% females). Participants will be randomly allocated to the HIIT (n= 16) or control groups (physical rest, n= 16). Participants will self-sample fasted dried blood spots for three consecutive days before the intervention (HIIT versus rest) to determine baseline sphingolipid levels. Dried blood spots will also be collected at five time points (2, 15, 30, 60min, and 24h) following the intervention (HIIT versus rest). To minimise the dietary influence, participants will receive a standardised diet for four days, starting 24 hours before the first dried blood sampling. For females, interventions will be timed to fall within the early follicular phase to minimise the menstrual cycle's influence on sphingolipid levels. Finally, physical activity will be monitored for the whole study duration using a wrist accelerometer.

**Ethics and dissemination: **The Ethics Committee of Northwest and Central Switzerland approved this protocol (ID 2022–00513). Findings will be disseminated in scientific journals and meetings.

**Trial Registration** The trial was registered on www.clinicaltrials.gov (NCT05390866, https://clinicaltrials.gov/ct2/show/NCT05390866) on May 25, 2022.

## Introduction

Cardiometabolic diseases represent a growing socioeconomic burden and concern for healthcare systems worldwide. ^1^^,^^2^ Improving cardiometabolic risk stratification should help clinicians better tailor prevention and treatment strategies to individual needs and thus combat this burden more effectively. ^3^ Currently, lipid profiling is still mainly limited to total cholesterol, low-density lipoprotein (LDL) and high-density lipoprotein (HDL) cholesterol, and triglycerides. ^4^ However, technological advances in mass spectrometry now allow to quantify less abundant circulating lipids such as sphingolipids. ^5^^–^^7^ Sphingolipids constitute a family of important bioactive lipids, which modulate numerous biological processes and are involved in the pathogenesis of coronary artery disease, type 2 diabetes mellitus (T2DM), and non-alcoholic fatty liver disease. ^8^^–^^10^ This suggests that comprehensive sphingolipid panels should be considered surrogates of cardiometabolic health. ^8^ Ceramides, the most studied sphingolipid class, have been shown to predict cardiovascular outcomes beyond LDL cholesterol. ^11^^–^^13^ Ceramide-based scores were more accurate than the 2019 SCORE of the European Society of Cardiology in terms of cardiovascular risk prediction ^14^^,^^15^ and are now used in clinical practice in the Mayo Clinic. ^16^^,^^17^ In addition, specific ceramide species were proved predictive for T2DM 10 years before the disease was diagnosed. ^18^ Mechanistically, excess caloric intake and inflammation stimulate sphingolipid synthesis. ^19^ Indeed, tumour necrosis factor-alpha (TNF-α) and free fatty acids activate sphingolipid synthesis enzymes. ^20^ Sphingolipids in excess are converted to ceramides, which accumulate on the surface of LDL, where they drive LDL transcytosis through the endothelium and uptake into macrophages. ^21^^–^^23^ This results in foam cell formation, vascular inflammation, and atherosclerosis. ^24^ Additionally, ceramides inhibit Akt/protein kinase B activity, which reduces the translocation of glucose transporters to the cell membrane, thereby contributing to peripheral insulin resistance and non-alcoholic fatty liver disease. ^25^^–^^27^ Thus, sphingolipid metabolism might become a future therapeutic target in clinical medicine, as demonstrated by rodent studies demonstrating a reduction of arterial hypertension, T2DM, atherosclerosis, and heart failure following ceramide synthesis inhibition. ^28^

**Figure 1.**
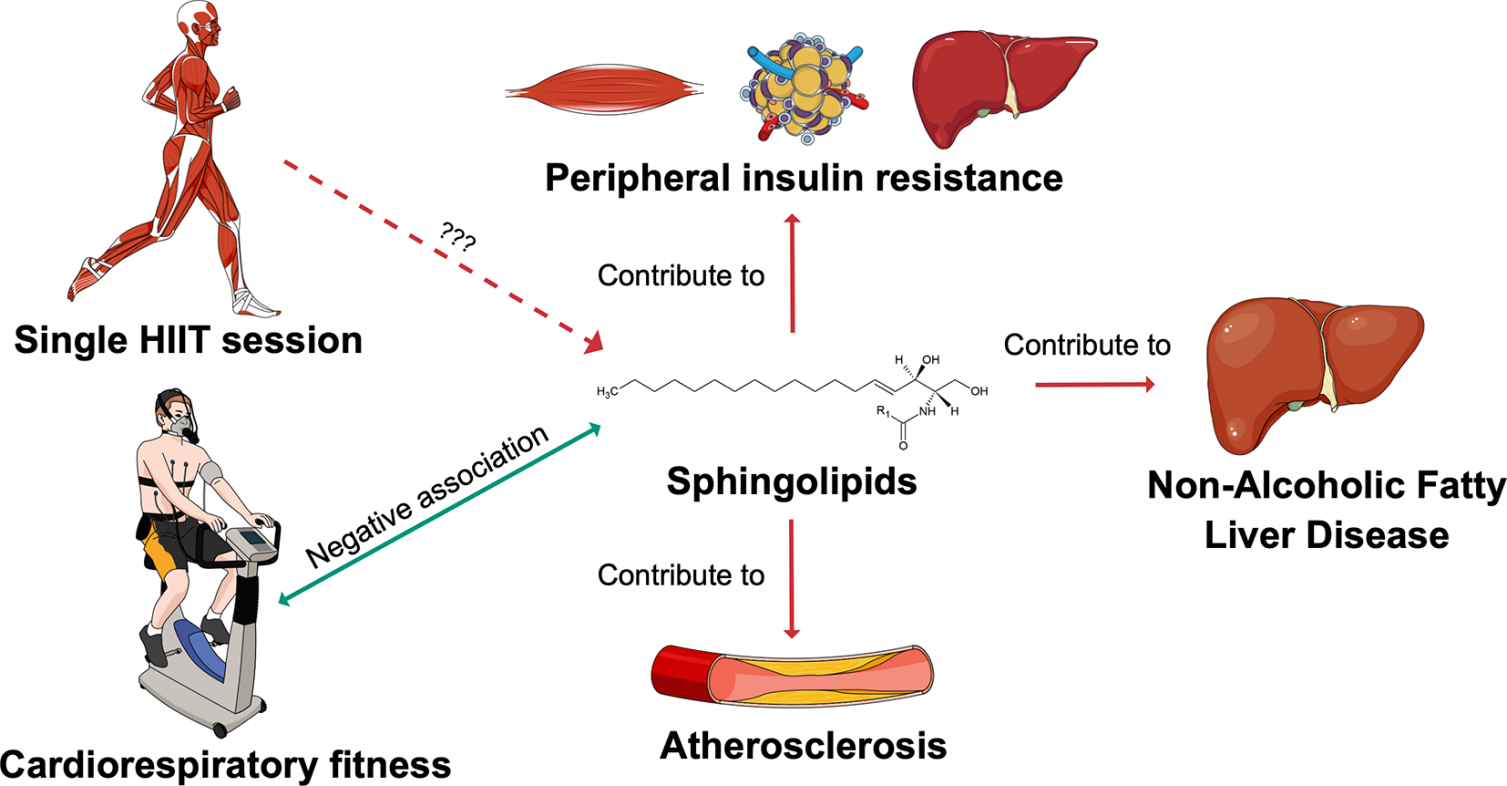
Sphingolipids as potential mediators of the exercise effects on cardiometabolic health. The SphingoHIIT study aims to investigate the effect of a single session of high-intensity interval training on circulating sphingolipids, which are novel biomarkers of cardiometabolic health. Abbreviations: HIIT = high-intensity interval training. This figure has been adapted with permission from Carrard, J. *et al.* A. How Ceramides Orchestrate Cardiometabolic Health—An Ode to Physically Active Living. *Metabolites* 2021, *11*, 675. https://doi.org/10.3390/metabo11100675

Regular physical activity is essential for maintaining general health ^29^^–^^31^ and for preventing and treating cardiovascular diseases, ^32^^,^^33^ insulin resistance, and T2DM. ^34^^,^^35^ Exercise mitigates traditional risk factors and directly improves cardiometabolic health by optimising vascular endothelial function, stimulating myokines secretion, and other mechanisms which remain to be elucidated. ^33^ Changes in sphingolipid metabolism might be one of the mechanisms through which exercise optimises cardiometabolic health ( Figure 1). This hypothesis is supported by the fact that circulating sphingolipids have been reported to be negatively associated with cardiorespiratory fitness (CRF). ^36^ Two preliminary studies investigated the effect of exercise on the circulating sphingolipidome. A single bout of moderate-intensity continuous training (MICT) increased circulating sphingolipids in endurance athletes, sedentary obese individuals, and patients with T2DM. ^37^ Conversely, 12 weeks of MICT in patients suffering from obesity or T2DM decreased plasma sphingolipid levels. ^38^ This duality could be mediated through inflammation as acute exercise tends to induce inflammation and regular exercise lowers it, while inflammation drives sphingolipid accumulation. ^19^^,^^39^

These preliminary studies investigated, however, a limited number of sphingolipid species (n=8 and 7, respectively), whereas targeted lipidomics has now matured into a high-throughput approach allowing for comprehensive analysis of lipid metabolism at the molecular species level. ^5^^,^^6^ This is particularly important as sphingolipids might have differential biological effects depending on acyl chain length and saturation, with shorter and unsaturated species being potentially more detrimental to human health. ^40^ Moreover, these studies examined the sphingolipid’s response to acute or regular MICT, while many studies showed that regular high-intensity interval training (HIIT) is a safe ^41^ and a more effective way to improve CRF ^42^^,^^43^ and insulin sensitivity. ^44^^,^^45^ In addition, the effect of HIIT on circulating sphingolipids might be more important than the one of MICT, as a single bout of HIIT is more intense than a single bout of MICT. ^46^^,^^47^ Furthermore, lipid profiles are potentially subject to day-to-day variations, an element not considered in these two studies. ^48^^–^^50^ Lastly, to investigate the physiological responses of sphingolipid species to exercise, it is necessary to examine healthy participants before investigating clinical populations, thereby avoiding confounding effects of chronic diseases on sphingolipid metabolism. To address these gaps, this randomised controlled trial will investigate the response of an extensive panel of circulating sphingolipids species to a single HIIT session within healthy individuals in their twenties while taking day-to-day variations into account. The results of this acute study will inform subsequent long-term exercise interventions. Moreover, investigating the effects of a single HIIT session is particularly relevant since signalling moieties, known as “exerkines”, are released in response to acute exercise. ^51^^,^^52^ These molecules can positively impact various organs through endocrine, paracrine, or autocrine signalling pathways. ^53^

## Protocol

### Aims and hypothesis

The present research project aims to assess, at the molecular species level, the response of the circulating sphingolipidome to a single HIIT session in healthy individuals aged 20-29. We hypothesise that circulating sphingolipid levels will temporarily increase following a single HIIT session, apart from the sphingosine-1-phosphate level, which might decrease. Indeed, this metabolite could be cardiometabolically favourable as patients with multiple sclerosis taking the drug fingolimod, a sphingosine-1-phosphate receptor modulator, experienced cardiac side effects such as first-dose bradycardia. ^54^

### Endpoints

The primary endpoints will be changes from pre- to post-intervention levels of the four most studied sphingolipid species, which are also included in the ceramide-based scores ( *i.e.* ceramide 16:0, ceramide 18:0, ceramide 24:0 and ceramide 24:1). ^14^ The secondary endpoints will be changes from pre- to post-intervention levels of the resting sphingolipid species to be acquired (n=57).

### Design and general considerations

This prospective two-arm, single-centre randomised controlled trial will be conducted at the Department of Sport, Exercise, and Health of the University of Basel. It will be carried out in accordance with the Declaration of Helsinki and the guidelines of Good Clinical Practice of the World Medical Association in 2013. The Ethics Committee of Northwest and Central Switzerland approved the study (project-ID 2022–00513). Substantial changes to the protocol will be submitted to the Ethics Committee for approval before implementation, as required by Swiss law. The study was registered on www.clinicaltrials.gov ( NCT05390866) on May 25, 2022, and follows the SPIRIT reporting guidelines. ^55^ All items from the World Health Organization Trial Registration Data Set are summarised in Table 1, according to the SPIRIT reporting guidelines. This is the first version of the protocol (22 November 2022). There will be no financial compensation for participation in the SphingoHIIT study.

**Table 1.** Items from the World Health Organization Trial Registration Data Set.

Data category	Information
Primary registry and trial identifying number	Clinicaltrials.gov, NCT05390866
Date of registration in primary registry	25 May, 2022
Secondary identifying numbers	SNCTP000004936, BASEC2022-00513
Source(s) of monetary or material support	*Freiwillige Akademische Gesellschaft Basel* (German for Voluntary Academic Society Basel, 14,400 Swiss francs) 2023 British Association of Sport & Exercise Medicine (BASEM) Research Bursary (5,000 pounds sterling)
Primary sponsor	Prof Arno Schmidt-Trucksäss, MD
Secondary sponsor(s)	n/a
Contact for public queries	Dr Justin Carrard, MD, Justin.carrard@unibas.ch
Contact for scientific queries	Dr Justin Carrard, MD, Justin.carrard@unibas.ch
Public title	The SphingoHIIT Study
Scientific title	Investigating the circulating sphingolipidome response to a single high-intensity interval training session (SphingoHIIT): Protocol for a randomised controlled trial
Countries of recruitment	Switzerland
Health condition(s) or problem(s) studied	Physiological Response of Sphingolipids to a Single HIIT Session
Intervention(s)	Intervention group: a single session of high-intensity interval training Control group: physical rest
Key inclusion and exclusion criteria	Ages eligible for study: 20-29 years Sexes eligible for study: both Accepts healthy volunteers: yes Inclusion and exclusion criteria: see Table 2
Study type	Interventional Allocation: randomised Intervention model: parallel assignment Masking: double Primary purpose: prevention
Date of first enrolment	1 September, 2022
Target sample size	32
Recruitment status	Data analysis
Primary outcome(s)	Concentration of circulating Cer16:0, Cer18:0, Cer24:0 and Cer24:1
Key secondary outcomes	Concentration of the resting circulating sphingolipid species to be acquired

The SPIRIT reporting guidelines recommends including these items in study protocols to provide a brief structured overview of a trial.

### Recruitment and eligibility criteria

This pilot study aims to include 32 healthy individuals aged 20-29 (50% females) from the Basel area (Switzerland) randomised to the HIIT or control group. Participants meeting the inclusion criteria ( Table 2) will be eligible for the study.

**Table 2.** Inclusion and exclusion criteria.

Inclusion criteria	Exclusion criteria
• Female or male sex. • Age = 20-29 years old. • BMI = 18.5-24.9 kg/m ^2^. • Meeting the WHO guidelines on physical activity, *i.e.* at least 150–300 minutes of moderate-intensity aerobic physical activity per week as well as muscle-strengthening activities on 2 or more days per week. • Clearance for physical activity according to the 2022 PAR-Q+. ^56^ • Regular menstrual cycle. • Informed consent as documented by signature.	• Females with known pregnancy or breastfeeding. • Females with known polycystic ovary syndrome. • Current exercise limiting conditions of the lower limbs (e.g., tendinopathy, fractures, or other musculoskeletal pathologies). • Known acute or chronic diseases: e.g. any active infectious diseases, past or current malignant tumours, lung diseases (e.g. bronchial asthma), cardiometabolic diseases (e.g. arterial hypertension, diabetes, dyslipidaemia), gastrointestinal diseases (e.g. coeliac disease, Crohn’s disease, ulcerative colitis), psychological disorders (e.g. depression, if medically diagnosed, anorexia, bulimia), endocrinological diseases (e.g. all types of diabetes mellitus, hyper- or hypothyroidism), nephrological diseases and neurological disorders. • Current or past smoking, current or past psychoactive drug use (alcohol excluded here, see below). ^57^ • Excessive alcohol consumption in the past two weeks, defined as either binge drinking (consuming five or more drinks during a single occasion) or heavy drinking (consuming 15 or more drinks per week). ^58^ • Current or regular medication use, including any kind of hormonal contraception. • Following diets such as: vegetarian, vegan, lactose-free and gluten-free, low-FODMAP (fermentable oligosaccharides, disaccharides, monosaccharides, and polyols). • Inability to follow the procedures of the study, e.g., due to linguistic or cognitive problems. • Concomitant or recent (last 4 weeks) involvement in another trial.

As regular physical activity is an essential component of global health, only participants meeting the WHO guidelines on physical activity will be included. Furthermore, being regularly physically active lower the risk of myocardial infarction and injuries during vigorous exercise. ^59^ Abbreviations: BMI = body mass index, WHO = World Health Organization, 2022 PAR-Q+ = 2022 Physical Activity Readiness-Questionnaire +.

Participants will be recruited through advertisements on the website of the Department of Sport, Exercise, and Health of the University of Basel and on the social media channels of the Department mentioned above. Detailed information about study procedures, risks, and benefits will be given by telephone to all participants. Following the phone call, all potential participants will be provided via e-mail with a participant information sheet and a consent form. All participants will have to sign a consent form and be informed about their right to withdraw from the study without consequences or the need for providing reasons.

### Group allocation

Participants will be randomly allocated either to the HIIT (n= 16, 50% females) or control (n= 16, 50% females) groups. Blocked randomisation will be used to reduce bias and achieve balance in allocating participants to both groups, as commonly done when the sample size is small. ^60^ The principal investigator will be responsible for the randomisation and will inform participants and investigators about the group allocation only on the day of the intervention ( *i.e.* opening of the sealed envelope). Therefore, participants and investigators supervising the intervention will be blinded for group allocation till the day of the intervention. The laboratory team in charge of the sphingolipid quantification will be blinded for group allocation.

### Study procedure

*Overview*

**Figure 2.**
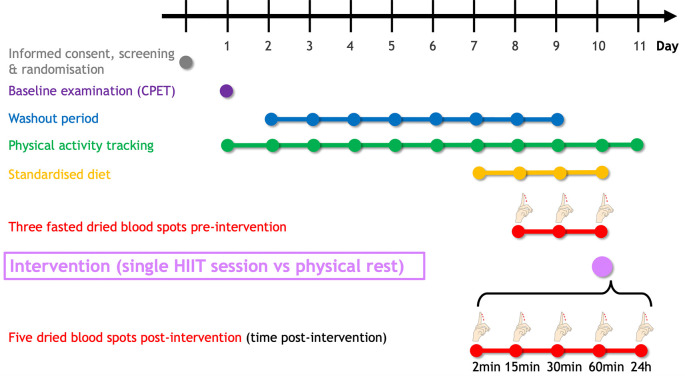
Timeline of the SphingoHIIT study. The SphingoHIIT study will last 11 days per participant. Following the baseline examination (which includes cardiopulmonary exercise testing), participants will have to avoid any vigorous-intensity physical activity (≥ 7 Metabolic Equivalent of Task) to maximise contrast between the pre-and post-intervention dried blood spots. To avoid potential confounding effects of food intake on circulating sphingolipids, participants will be fed starting 24h before the first dried blood spot collection. Abbreviations: CPET = cardiopulmonary exercise testing, HIIT = high-intensity interval training

Figure 2 summarises the planned study procedure. Following eligibility criteria assessment, a maximal cardiopulmonary exercise testing (CPET) will be performed to determine peak oxygen uptake ( $V˙O_{2}$peak), heart rate, and power output. An eight-day washout period will be carried out until the intervention (HIIT vs physical rest). In the three days preceding the intervention, participants will self-sample fasted dried blood spots (DBS) to determine sphingolipid baseline levels. DBS will be collected at five additional fixed time points (2min, 15min, 30min, 60min, and 24h) following the intervention. To minimise the dietary influence, participants will be asked to uniquely consume the provided individualised, pre-packaged meals starting one day before the first dried blood sampling. The study will last 11 days for each participant.

*Eligibility screening*

Potential participants will be screened for initial eligibility during a first in-person visit at the Department of Sport, Exercise, and Health of the University of Basel, which will take place after potential participants return the signed consent form. Inclusion and exclusion criteria will be carefully reviewed during the first in-person visit. Height and weight will be measured, and body mass index (BMI) will be calculated.

*Baseline clinical assessment (day 1)*

Participants included after the eligibility screening will be invited for a clinical baseline examination. Physical examination and vital sign measurements (blood pressure, heart rate, respiration rate, oxygen saturation, and body temperature) will be conducted. Body composition will be analysed by four-segment bioelectrical impedance analysis using the InBody 720 (Inbody Co. Ltd., Seoul, South Korea). InBody 720 provides reliable body composition measurements compared to dual-energy x-ray absorptiometry analysis. ^61^^,^^62^ Before the measurement, participants must refrain from intense physical activity for 24 h, fast for a minimum of two hours, and void their bladder. Physical activity will be objectively monitored throughout the whole study duration (11 days) using a wrist-worn triaxial accelerometer (GeneActiv Activinsights Ltd., Kimbolton, UK). The device will be attached to the participant’s nondominant wrist and worn day and night continuously in their free-living conditions. Accelerometry data will be exported using the GENEActiv software version 3.2 (GeneActiv Activinsights Ltd. Kimbolton, UK). Participants will receive an oral and written introduction to the accelerometer’s use.

A resting ECG will also be performed to rule out cardiac contraindications to maximal exertion. A maximal cardiopulmonary exercise testing (CPET) will be conducted on a cycle ergometer to determine $V˙O_{2}$peak, peak heart rate, and peak power output (Ergoselect 200; Ergoline, Bitz, Germany). A 3-min warm-up will be performed either unloaded or with a load of 10, 20, 50, or 50 W for protocols 1 to 5, respectively. The warm-up will be followed by a ramp protocol 1-5 with a workload increase of 7, 10, 15, 20, or 30 Watts/min, respectively. The 3-min recovery phase will be performed on the same wattage as the warm-up. The protocol will be chosen based on the following formula by Hansen *et al.* ^63^ The increment closest to the one evaluated with the formula will be used.

$$\text{Work rate increment}\;\left(W/\min\right)=V˙O_{2}\text{peak}–V˙O_{2}\text{unloaded}/100,$$

$$V˙O_{2}\text{peak}\;\left(\mathrm{ml}/\min\right)\;\mathrm{men}=\text{height}\;\left(\mathrm{cm}\right)–\mathrm{age}\;{\left(\text{year}\right)}^{*}\;20,$$

$$V˙O_{2}\text{peak}\;\left(\mathrm{ml}/\min\right)\;\text{women}=\text{height}\;\left(\mathrm{cm}\right)–\mathrm{age}\;{\left(\text{year}\right)}^{*}\;14,$$

$$V˙O_{2}\text{unloaded}\;\left(\mathrm{ml}/\min\right)=150+\left(6^{*}\;\text{weight}\;\left(\mathrm{kg}\right)\right).$$

Participants will receive the HemaXis DB10 whole blood collection device (DBS System SA, Gland, Switzerland) for at-home capillary whole blood sampling. To ensure the proper performance of blood sampling, they will receive written and oral instructions on the HemaXis DB10 device.

*Washout period*

A washout period (days 2 to 9) will take place between the CPET and the intervention (HIIT versus rest), during which participants will be asked not to perform any vigorous-intensity physical activity (≥ 7 Metabolic Equivalent of Task).

*Pre-intervention blood sampling*

From day 8 to day 10, participants will self-sample two DBS in a fasting state between 6 and 8 am to assess baseline values and day-to-day variations of the circulating sphingolipid species levels. For each time point of blood sampling, two DBS will be sampled. The same blood source will provide the two DBS so that only one skin puncture per blood sample will be necessary, as two droplets of blood suffice for two DBS. Participants will be instructed to let the blood spot dry for 10 minutes, put the DBS in a provided plastic bag containing a small silica gel bag to absorb humidity and store them in their fridge at around 4°C. On day 10, they will bring back the DBS from days 8-10. There is no need to refrigerate the samples for transport when bringing the samples from home to the Department of Sports, Exercise, and Health of the University of Basel.

*Exercise intervention (day 10)*

A single HIIT session will be performed on day 10 between 6 and 8 am. It will consist of a 3-min warm-up, followed by four 4-min intervals performed at 85-95% of peak heart rate, interspersed with 3-min of active recovery periods at the rating of perceived exertion (RPE) 11-13 ( *i.e.*, fairly light to somewhat hard). ^64^ A 2-min cool-down will follow the HIIT. This protocol was chosen because it has been extensively studied in healthy and clinical populations and its effects on CRF improvement are well-documented. ^42^^,^^65^ This protocol also fulfilled the requirements of a high-volume HIIT. ^66^ The guidelines for HIIT prescription and monitoring established by Taylor *et al.* will be strictly followed in this study. ^42^ Exercise professional supervising the session will record heart rate (using a heart rate monitor), power output (read on the ergometer) and RPE within the final 15 seconds of each minute. These data will then be used to assess adherence to the prescribed intensity.

*Control intervention (day 10)*

The control group participants will undertake the same procedures except for the HIIT session. Participants of the control group will have to physically rest in the lab in a seated position for the duration of the HIIT (30 min) before post-intervention DBS can be collected to ensure similar daily times for sample collection between both groups. During the rest period, participants are allowed to read a book and use a smartphone or a computer.

*Post-intervention blood sampling*

Two DBS will be collected at 2 min, 15 min, 30 min, 60 min (directly in the lab), and 24 h post-intervention ( *i.e.*, DBS of day 11, at home). DBS from day 11 will be returned on day 11.

*Sphingolipidomics*

All DBS will be vacuumed and subsequently stored at -80°C at the Department of Sport, Exercise, and Health of the University of Basel before being delivered to the Metabolomics Unit of the Faculty of Biology and Medicine at the University of Lausanne. To quantify an extensive panel of circulating sphingolipids (n=61), a high-coverage method using reversed-phase liquid chromatography coupled to tandem mass spectrometry (RPLC-MS/MS) will be applied, as previously described. ^7^

*Standardised diet*

To minimise the differential influence of food intake on sphingolipid levels between participants, each participant will be provided with individualised, pre-packaged meals for days 7 to 10. The meals will have to be consumed during predetermined time windows, *i.e.*, between 7 and 9 am for breakfast (but after dried blood sampling in any case), between 11 am and 1 pm for lunch, between 4 and 5 pm for an afternoon snack and between 6.30 and 8.30 pm for dinner. All participants will be fed to energy balance to maintain weight stability throughout the study period. Energy requirements will be calculated with the formulas of Mifflin St. Joer ^67^ and the National Institute of Diabetes and Digestive and Kidney Diseases (NIDDK) Body Weight Planner. ^68^^,^^69^ All diets will contain ~55% energy from carbohydrates, ~25% energy from fat, and ~20% energy from protein. To monitor diet adherence, participants will be instructed to return all non-consumed foods from the pre-packaged meals to the lab and take photos of additionally consumed foods for later analysis of food and energy intake. Participants will also be asked to refrain from alcohol consumption during the dietary control period and from caffeine intake before the HIIT on day 10.

*Controlling for menstrual cycle*

For females, the regularity of the menstrual cycle will be assessed during the screening procedure using items five and seven of the Reproductive Status Questionnaire for Menstrual Cycle Studies. ^70^ The goal will be to time the study, so that day 8 coincides with the beginning of the early follicular phase. This phase is indicated by the onset of bleeding and corresponds to the lowest concentrations of oestrogen and progesterone. ^71^ Combined with the exclusion of females taking any hormonal contraceptives, this will minimise the effect of oestrogen and progesterone on sphingolipid levels and reduce heterogeneity among female participants. ^71^

### Sample size calculation

**Figure 3.**
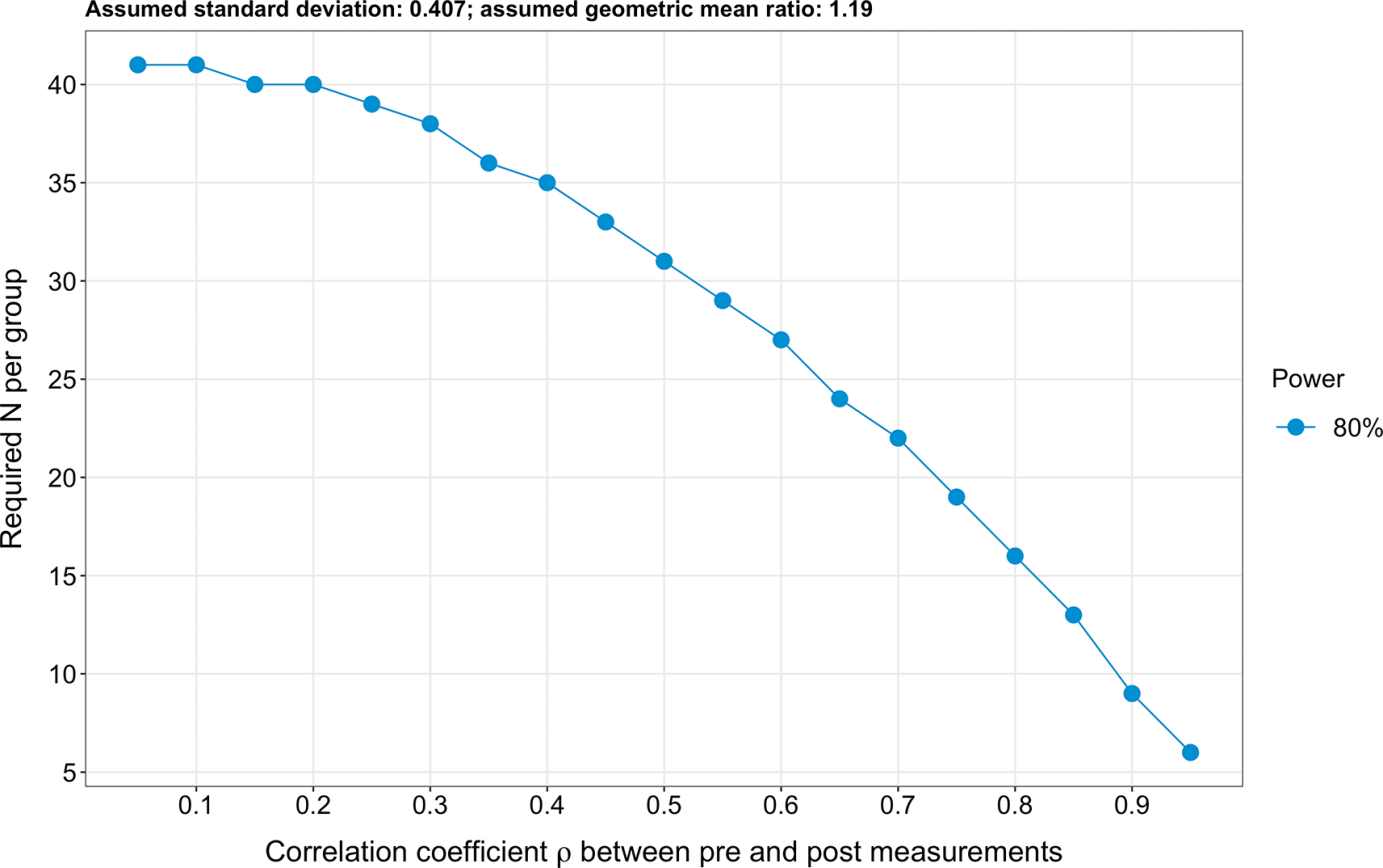
Illustrated sample size calculation. A sample size of 16 participants per group was obtained (expressed as a detectable geometric mean ratio), assuming a power of 80%, an effect size of 1.19 (expressed as a geometric mean ratio), a standard deviation of 0.407 and a correlation coefficient ρ between pre-and post-intervention values of 0.8.

Due to the extensive number of targeted endpoints (n=61), a classical sample size calculation cannot be performed. Therefore, to estimate the required sample size, we based our analysis on the four most investigated sphingolipid species, which are also the species entering the ceramide scores used at the Mayo Clinic ( *i.e.* ceramide 16:0, ceramide 18:0, ceramide 24:0 and ceramide 24:1). ^16^^,^^17^ Further, we hypothesised for simplicity that log2-transformed pre-intervention sphingolipid levels are similar in both the intervention and the control groups (due to the randomisation). Next, we assumed a standard deviation of log2-transformed sphingolipid levels of 0.407, an effect size (expressed as a geometric mean ratio) of 1.19 and a correlation coefficient between pre-and post-intervention values of 0.8. To obtain these values, we used the raw data of the four sphingolipids mentioned above issued from a previous exercise intervention study conducted by Bergman *et al*. ^37^ Specifically, we calculated the geometric mean of the sphingolipid concentrations post- and pre-exercise for the four primary endpoints (i.e. ceramide 16:0, ceramide 18:0, ceramide 24:0 and ceramide 24:1). We then calculated the ratio of the geometric means post- to pre-exercise. Finally, we averaged the geometric mean ratios obtained for these four endpoints and obtained a value of 1.194. We then log2-transformed 1.194 and obtained 1.19. Therefore, 1.19 is the log2-transformed geometric mean ratios of the post- to pre-exercise sphingolipid concentrations. In other words, the geometric means of the sphingolipid concentrations differ by a factor of 1.19 post- to pre-exercise in healthy individuals in the study conducted by Bergman *et al.* ^37^ Finally, we analysed covariance (ANCOVA) to calculate the sample size. We obtained a result of 16 participants per group for a power of 80% ( Figure 3). Lastly, it should be pointed out that the planned repeating measurements will enhance precision. ^72^ The R code used to calculate the sample size is freely available on Open Science Framework. ^73^

### Statistical analyses

**Figure 4.**
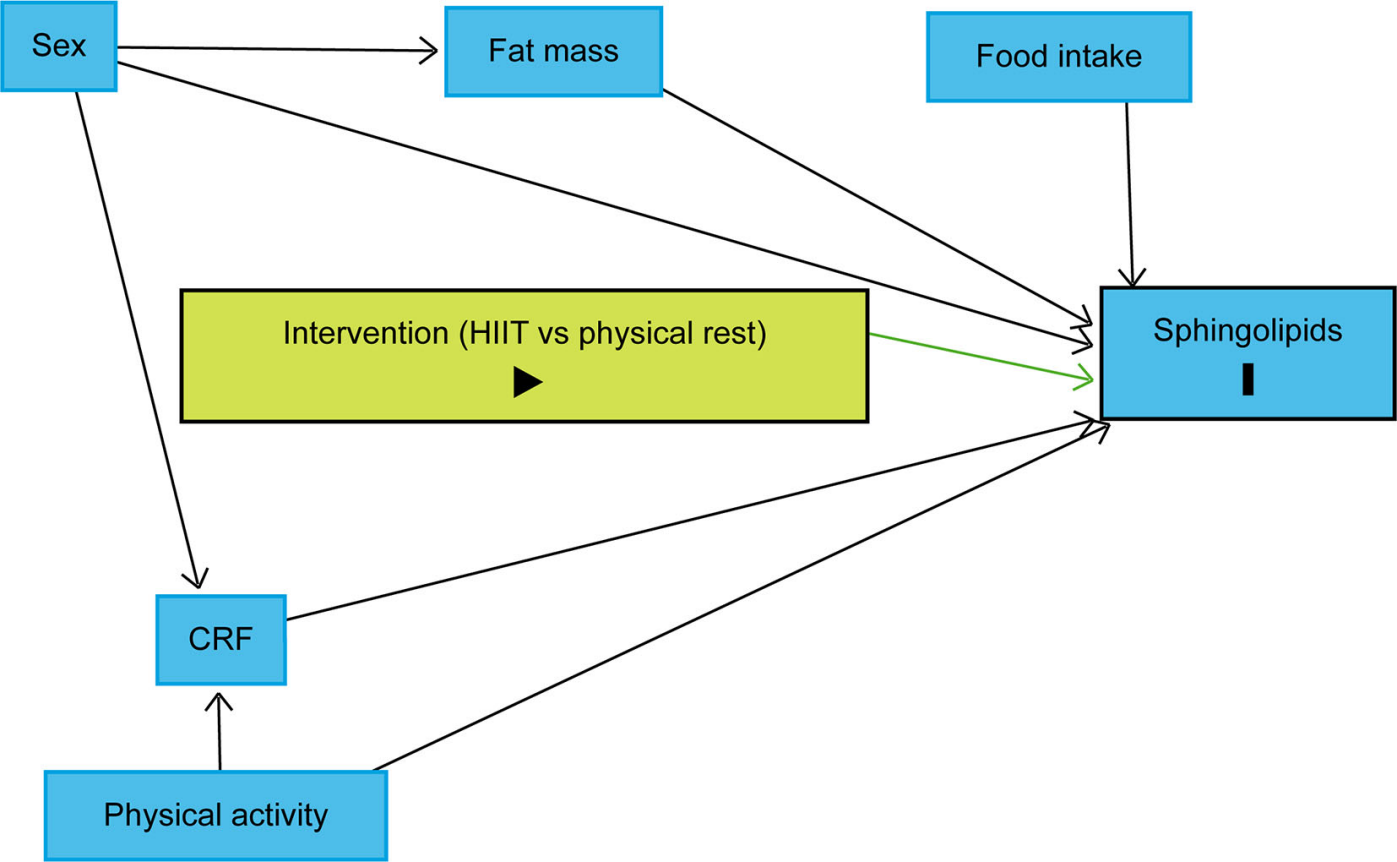
Directed acyclic graph representing the intervention effect on sphingolipid levels. The intervention (HIIT vs physical rest) is defined as the exposure and sphingolipids as the outcomes. Due to the randomisation, the exposure has no ancestor. Thus, no variable influences simultaneously the exposure and outcomes, which implies that there is no confounding variable. Sex, body fat mass, CRF, and physical activity were identified as variables to be included in the statistical models to reduce the outcome variation and improve the precision of the average causal effect of the intervention on the sphingolipidome. Abbreviations: HIIT = high-intensity interval training, CRF = cardiorespiratory fitness, black triangle pointing to the right on green background = exposure, black bold vertical bar = outcomes, blue background = variables to be included in the statistical models to reduce the outcome variation and improve the precision of the average causal effect of the intervention. The figure was realised with http://www.dagitty.net.

The trajectory over time of each lipid species will be modelled using linear mixed models with a random effect for each subject. To identify variables, we should include in the models, we drew a causal directed acyclic graph (DAG) using DAGitty. ^74^ Sex, body fat mass, CRF, and habitual physical activity were identified as variables to be included in the linear mixed models to reduce the outcome variation and improve the precision of the average causal effect of the intervention on the sphingolipidome ( Figure 4). ^72^ As explained above, the design will control food intake as each participant will be provided with individualised, pre-packaged meals for days 7 to 10. It is also assumed that age difference will not play a significant role as all participants will be between 20 and 29 years old. Therefore, the models will include fixed effects for time points, sex, fat body mass, CRF, physical activity, and a three-way interaction term between the group, time point and an indicator variable for time points following the intervention. Planned contrasts between the groups at each time point following the intervention will allow assessing differences in lipid concentrations between the groups after the intervention. P-values and confidence intervals will be adjusted for multiple testing using the Benjamini-Hochberg method. ^75^ We will use residual diagnostic plots to assess whether the model assumptions are met. If model assumptions are violated, lipid concentrations will be log-transformed. If the proportion of missing data is below 5%, a complete case analysis will be done. Otherwise, we will consider multiple imputations. Drop-outs will be replaced by recruiting new subjects to achieve the targeted sample size of 32 participants.

The significance level is set at α = 0.05, and all tests will be two-sided. All analyses will be done according to the intention-to-treat principle. Statistical analyses will be conducted using R (version 4.0.2 or later). Study results will be reported in compliance with the CONSORT statement. ^76^

### Data management

Data will be pseudonymised, stored on the protected server of the University of Basel and accessible only for authorised personnel to fulfil the research objectives described in the present protocol. Biological material collected during the SphingoHIIT study will be stored at the Department of Sport, Exercise and Health for 10 years after study termination.

### Adverse event

Any adverse event will be classified, and its severity assessed by the investigator according to the guidelines of the International Council for Harmonisation of Technical Requirements for Pharmaceuticals for Human Use. ^77^ The Ethics Committee will be informed in due time as required by Swiss law.

### Dissemination

Findings and data will be disseminated in scientific journals and meetings.

### Study status

Data analysis.

## Discussion

Optimising patients’ metabolic risk stratification in clinical practice has the potential to improve personalised prevention and early treatment of cardiometabolic diseases. Sphingolipids in general, and ceramides in particular, are essential bioactive lipids and promising biomarkers to enhance patients’ phenotyping and pre-symptomatic management of cardiometabolic disorders. This preliminary RCT aims to reveal the physiological impact of an acute bout of high-intensity exercise on the circulating sphingolipidome in healthy individuals in their twenties, thereby avoiding the confounding effects of chronic diseases on sphingolipid metabolism. In addition, the brevity of this RCT enables us to strictly control nutrition, physical activity levels, and the menstruation cycle. Thus, this well-controlled setting will allow for robust effect estimates and subsequent power calculations for further studies aiming to investigate the effect of regular exercise on the sphingolipid profile in different clinical populations.

Investigating sphingolipid responses to a single HIIT session on the molecular species level could also highlight novel ways through which exercise orchestrates cardiometabolic health. ^19^ While it is well established that regular exercise improves overall health, the mechanisms underlying exercise-mediated health benefits remain indeed ill-defined. ^52^ As acute exercise releases signalling moieties (“exerkines”) that mediate short- and long-term effects of physical activity on the human body, ^53^ this RCT may identify key sphingolipid species related to short-term exercise adaptation and ultimately pave the way for novel health-monitoring strategies. Altogether, improving the molecular understanding of exercise medicine will further contribute to establishing this novel medical discipline as a cornerstone in preventing and treating cardiometabolic disorders.

## Author contributions

This work has been developed with the contribution of each co-author. The manuscript underwent several revisions, with substantial contributions provided by each co-author. All authors provided critical feedback and have read and approved the final manuscript.

Conceptualisation: JC, TA, NW, IC, CS, HH, HGA, JI, and AST. Funding acquisition: JC and AST. Methodology: JC, TA, NW, JB, DI, LS, IC, TH, CH, KK, HGA, JI, and AST. Project administration: JC and TA. Resources: JC, CH, KK, JI, and AST. Software: JC and DI. Supervision: JC and AST. Visualisation: JC, JB, and DI. Writing - Original Draft Preparation: JC, TA, NW. Writing - Review & Editing: JC, TA. NW, JB, DI, LS, TH, IC, CS, HGA, CH, KK, HH, JI, and AST.

## Data Availability

The R code used to calculate the sample size is freely available on Open Science Framework, https://doi.org/10.17605/OSF.IO/53QND. ^73^ Open Science Framework: SPIRIT checklist for “Investigating the circulating sphingolipidome response to a single high-intensity interval training session (SphingoHIIT): Protocol for a randomised controlled trial”, https://doi.org/10.17605/OSF.IO/53QND. ^73^ Data are available under the terms of the Creative Commons Attribution 4.0 International license (CC-BY 4.0).
