# Supplementary material for: Investigating the circulating sphingolipidome response to a single high-intensity interval training session within healthy females and males in their twenties (SphingoHIIT): Protocol for a randomised controlled trial
Source: F1000Res. 2023 Aug 18;11:1565. Originally published 2022 Dec 22. [Version 3] doi: 10.12688/f1000research.128978.3 (PMC10390797; doi:10.12688/f1000research.128978.3)
Supplement: Peer review history

# Peer review: Reviewer response for version 2

**DOI:** 10.5256/f1000research.152117.r187805

**Published:** 2023-07-31

**Authors:** Boris Schmitz

**Affiliations:** 1 Department of Rehabilitation Sciences, Faculty of Health, Witten/Herdecke University, Witten, North Rhine-Westphalia, Germany

Carrard *et al.* provide a study protocol entitled, "Investigating the circulating sphingolipidome response to a single high-intensity interval training session within healthy females and males in their twenties (SphingoHIIT): Protocol for a randomised controlled trial". The planned investigation is of great interest and adds to the scientific knowledge in the field. The protocol is well-written, and the presentation is clear and concise. I have only minor comments, some of them might be obsolete since the study status appears to be "data analysis".

- Blinding: It would be beneficial if sample analysis could be blinded.
- Physical activity prior to the study: How will this be assessed? I assume by questionnaire?
- Exclusion criteria: should formally also include rare diseases affecting ceramides (i.e. Fabry disease).
- Exercise Intervention: It could be of interest to also sample for lactate at the end of the HIIT.
- Data management: Please replace “anonymized” by “pseudonymized”.
- It might be helpful to add one or two sentences on how one session of HIIT (as planned) might differ in effects on sphingolipids from regular physical exercise, as this is mentioned frequently in the paper. Also, the sentence "Additionally, this study may…open new horizons for creating safe sphingolipid-lowering pharmaceuticals" might be a bit of an overstatement.

I wish the authors the best of luck with their study and look forward to the results.

Is the study design appropriate for the research question?

Yes

Is the rationale for, and objectives of, the study clearly described?

Yes

Are sufficient details of the methods provided to allow replication by others?

Yes

Are the datasets clearly presented in a useable and accessible format?

Not applicable

Reviewer Expertise:

Exercise Physiology; Rehabilitation Medicine

I confirm that I have read this submission and believe that I have an appropriate level of expertise to confirm that it is of an acceptable scientific standard.

# Response

**Published:** 2023-08-13

**Authors:** Justin Carrard

**Affiliations:** University of Basel, Switzerland

Dear Dr Schmitz,

 We would like to thank you very much for your positive and insightful comments. These have been addressed point-by-point (below) and in the revised manuscript via track changes. We hope that you will agree that these revisions have further strengthened our manuscript.

 Yours sincerely,

 The authors

**Carrard et al. provide a study protocol entitled, "Investigating the circulating sphingolipidome response to a single high-intensity interval training session within healthy females and males in their twenties (SphingoHIIT): Protocol for a randomised controlled trial". The planned investigation is of great interest and adds to the scientific knowledge in the field. The protocol is well-written, and the presentation is clear and concise.**

 Thank you for your positive and encouraging comments.

**I have only minor comments, some of them might be obsolete since the study status appears to be "data analysis".**

  

- **Blinding: It would be beneficial if sample analysis could be blinded.**

The laboratory team in charge of the sphingolipid quantification is blinded for group allocation (HIIT vs physical rest), as they received the samples and their related IDs but not the group allocation.

 The information was added to the manuscript.

- **Physical activity prior to the study: How will this be assessed? I assume by questionnaire?**

Physical activity prior to the study was assessed orally during eligibility screening by asking the following three questions:

- How many minutes of moderate-intensity physical activity do you do on average per week?
- How many minutes of vigorous-intensity physical activity do you do on average per week?
- How many minutes of muscle-strengthening activity do you do on average per week?

- **Exclusion criteria: should formally also include rare diseases affecting ceramides (i.e. Fabry disease).**

Thank you for this important comment. We agree that we could have specifically mentioned rare diseases affecting ceramide metabolism in the exclusion criteria. However, these diseases lead to reduced exercise capacity and are thus exercise-limiting (1). Therefore, diseases affecting ceramide metabolism are encompassed in the exclusion criteria “current exercise limiting conditions of the lower limbs” and “known acute or chronic diseases”.

 Since data collection is now completed, we decided not to modify exclusion criteria a posteriori.

- **Exercise Intervention: It could be of interest to also sample for lactate at the end of the HIIT.**

Thank you for this comment. We agree that it could have been of interest to measure lactate directly after the HIIT. As explained above, data collection is now completed. Therefore, we cannot implement this measurement anymore.

 During and at the end of the HIIT session, we monitored heart rate (bpm), power output (W) and the rating of perceived exertion (RPE) using the original Borg scale (6 to 20).

 The fact that power output also was monitored (in addition to heart rate and RPE) was added to the manuscript.

- **Data management: Please replace “anonymized” by “pseudonymized”.**

We rewrote it as suggested.

- **It might be helpful to add one or two sentences on how one session of HIIT (as planned) might differ in effects on sphingolipids from regular physical exercise, as this is mentioned frequently in the paper.**

We hypothesised that an acute bout of exercise would lead to a short-term increase in circulating sphingolipid levels (2). As a single bout of high-intensity interval training (HIIT) is more intense than a single bout of moderate-intensity continuous training (MICT) (3), we assumed that the effect of HIIT on circulating sphingolipids might be more important than the one of MICT (4). Therefore, we opted for a single bout of HIIT as an intervention for the present study.

 Regular physical exercise (of HIIT or MICT nature) might lead to a reduction in circulating sphingolipid levels (5). We will test this hypothesis in a subsequent study.

 This is summarised in the second paragraph of the introduction:

 “Two preliminary studies investigated the effect of exercise on the circulating sphingolipidome. A single bout of moderate-intensity continuous training (MICT) increased circulating sphingolipids in endurance athletes, sedentary obese individuals, and patients with T2DM. ^37^ Conversely, 12 weeks of MICT in patients suffering from obesity or T2DM decreased plasma sphingolipid levels. ^38^ This duality could be mediated through inflammation as acute exercise tends to induce inflammation and regular exercise lowers it, while inflammation drives sphingolipid accumulation. ^19^^ ,^^39^ “

 We added, in the third paragraph of the introduction, the following sentence to better explain the choice of HIIT over MICT:

 “In addition, the effect of HIIT on circulating sphingolipids might be more important than the one of MICT, as a single bout of HIIT is more intense than a single bout of MICT. ^76, 77^”

- **Also, the sentence "Additionally, this study may…open new horizons for creating safe sphingolipid-lowering pharmaceuticals" might be a bit of an overstatement.**

Thank you for this comment. We removed this sentence, as you kindly suggested.

**I wish the authors the best of luck with their study and look forward to the results.**

 Thank you for your insightful review.

**References**

 1.         Powell AW, Jefferies JL, Hopkin RJ, Mays WA, Goa Z, Chin C. Cardiopulmonary fitness assessment on maximal and submaximal exercise testing in patients with Fabry disease. Am J Med Genet A. 2018;176(9):1852-7.

 2.         Bergman BC, Brozinick JT, Strauss A, Bacon S, Kerege A, Bui HH, et al. Serum sphingolipids: relationships to insulin sensitivity and changes with exercise in humans. American Journal of Physiology-Endocrinology and Metabolism. 2015;309(4):E398-E408.

 3.         Falz R, Fikenzer S, Holzer R, Laufs U, Fikenzer K, Busse M. Acute cardiopulmonary responses to strength training, high-intensity interval training and moderate-intensity continuous training. European Journal of Applied Physiology. 2019;119(7):1513-23.

 4.         Rahmati-Ahmadabad S, Azarbayjani MA, Farzanegi P, Moradi L. High-intensity interval training has a greater effect on reverse cholesterol transport elements compared with moderate-intensity continuous training in obese male rats. Eur J Prev Cardiol. 2021;28(7):692-701.

 5.         Kasumov T, Solomon TP, Hwang C, Huang H, Haus JM, Zhang R, et al. Improved insulin sensitivity after exercise training is linked to reduced plasma C14:0 ceramide in obesity and type 2 diabetes. Obesity (Silver Spring). 2015;23(7):1414-21.

# Peer review: Reviewer response for version 2

**DOI:** 10.5256/f1000research.152117.r182656

**Published:** 2023-06-30

**Authors:** Stefan Gross

**Affiliations:** 1 German Centre for Cardiovascular Research (DZHK), Greifswald, Germany; 2 Dept. of Cardiology, University Medicine Greifswald, Greifswald, Germany

Dear Dr Carrard

 Thank you very much for your answers to my initial comments. Everything is properly addressed and now completely clear.

 I wish you good luck successfully conducting your study and I am looking forward to seeing your published study results.

Is the study design appropriate for the research question?

Yes

Is the rationale for, and objectives of, the study clearly described?

Yes

Are sufficient details of the methods provided to allow replication by others?

Partly

Are the datasets clearly presented in a useable and accessible format?

Not applicable

Reviewer Expertise:

More than 10 years of cardiovascular epidemiology incl. exercise testing, biomarker research and sphingolipids/ceramides. More than 20 years of experience with statistical methods especially regression methods, sample size calculation etc.

I confirm that I have read this submission and believe that I have an appropriate level of expertise to confirm that it is of an acceptable scientific standard.

# Peer review: Reviewer response for version 1

**DOI:** 10.5256/f1000research.141622.r178102

**Published:** 2023-06-22

**Authors:** Stefan Gross

**Affiliations:** 1 German Centre for Cardiovascular Research (DZHK), Greifswald, Germany; 2 Dept. of Cardiology, University Medicine Greifswald, Greifswald, Germany

The manuscript of Carrard and colleagues describes the study protocol for an investigation on the relationship between S1p and exercise training and whether this biomarker is modified by exercise training. It is a pilot RCT. The rationale, study plan and also statistical plans are convincing in general. I highly appreciate that the authors also utilized DAGs for determining possible analysis adjustments. The whole manuscript is written in a comprehensive, straightforward way and easy to follow by the reader.

 However, I still have some minor remarks that should be addressed:

1. **Sample size calculation:** please provide additional details / information on the utilized software packages and if possible also the utilized code. It is not clear to me, whether the assumed effect size of 1.19 is only the post-treatment geometric group mean ratio? Or is it the assumed ratio between the pre-post changes of both groups? I tried to recapitulate the calculation made here and I always reached larger group sizes than 16.
2.
**Statistical analysis** **: **Since this is a randomized trial you should not include into the linear mixed effects model a main effect for the treatment group. The treatment group should be only included in the interaction term with timepoints itself. Randomization implies there is no group difference at baseline pre treatment, so both groups have the same intercept. Treatment can only causally act after the baseline timepoint.
3. Just as a minor remark, sphingolipids and ceramides are presented in the introduction only as having detrimental effects especially in the cell/molecular mechanism part. The literature is not clear so far to my knowledge e.g. how chain length of ceramides are related to CRF and CV risk. It seems that short chain ceramides have more "detrimental" effects while very long chain ceramides seem to have also "positive" effects. Saturation seems to play also a role: C24:0 is more positively associated with CRF and inversely with CVD mortality while C24:1 seems to be different. C16/C24 ratio is inversely associated with mortality and risk of CV incident events. See e.g. Peterson *et al.,* (2018) ^1^. So maybe include 1-2 sentences into the introduction or discussion that the mechanistic picture is rather complex and it depends about which sphingolipid/ceramide in terms of chain-length and saturation we are speaking.

After minor revision it should be accepted and I wish the authors good luck with conducting the study. I am highly looking forward to the upcoming and interesting results of this study.

Is the study design appropriate for the research question?

Yes

Is the rationale for, and objectives of, the study clearly described?

Yes

Are sufficient details of the methods provided to allow replication by others?

Partly

Are the datasets clearly presented in a useable and accessible format?

Not applicable

Reviewer Expertise:

More than 10 years of cardiovascular epidemiology incl. exercise testing, biomarker research and sphingolipids/ceramides. More than 20 years of experience with statistical methods especially regression methods, sample size calculation etc.

I confirm that I have read this submission and believe that I have an appropriate level of expertise to confirm that it is of an acceptable scientific standard, however I have significant reservations, as outlined above.

## References

[1] : Ceramide Remodeling and Risk of Cardiovascular Events and Mortality. *J Am Heart Assoc* .2018;7(10) : 10.1161/JAHA.117.007931 10.1161/JAHA.117.007931 29728014PMC6015315

# Response

**Published:** 2023-06-25

**Authors:** Justin Carrard

**Affiliations:** University of Basel, Switzerland

Dear Dr Gross,

 We would like to thank you very much for your positive and insightful comments ( **in bold below**). These have been addressed point-by-point (below) and in the revised manuscript via track changes. We hope that you will agree that these revisions have further strengthened our manuscript.

 Yours sincerely,

 The authors

**The manuscript of Carrard and colleagues describes the study protocol for an investigation on the relationship between S1p and exercise training and whether this biomarker is modified by exercise training. It is a pilot RCT. The rationale, study plan and also statistical plans are convincing in general. I highly appreciate that the authors also utilized DAGs for determining possible analysis adjustments. The whole manuscript is written in a comprehensive, straightforward way and easy to follow by the reader.**

 Thank you for your positive and encouraging comments.

**However, I still have some minor remarks that should be addressed:**

**Sample size calculation: please provide additional details / information on the utilized software packages and if possible also the utilized code. It is not clear to me, whether the assumed effect size of 1.19 is only the post-treatment geometric group mean ratio? Or is it the assumed ratio between the pre-post changes of both groups? I tried to recapitulate the calculation made here and I always reached larger group sizes than 16.**

 We used the software R (version 4.0.2 or later) and the following packages: emmeans, lme4, nlme, ggplot2, tidyverse, visreg, and readxl. The R-code is now freely available on Open Science Framework ( https://osf.io/53qnd?mode=&revisionId=&view_only=).

 To obtain 1.19, we first asked Bergman et al. if they would agree to share with us the circulating sphingolipid values pre- and post-exercise for the healthy participants of their studies (1). They kindly agreed to do so. Kindly note that we are, however, not authorised to publish these data along our study protocol as we do not own these data. We then calculated the geometric mean of the sphingolipid concentrations post- and pre-exercise for the four primary endpoints ( *i.e.* ceramide 16:0, ceramide 18:0, ceramide 24:0 and ceramide 24:1) before we calculated the ratio of the geometric means post- to pre-exercise. Finally, we averaged the geometric mean ratios obtained for these four endpoints and obtained a value of 1.194. We then log2-transformed 1.194 and obtained 1.19. Therefore, 1.19 is the log2-transformed geometric mean ratios of the post- to pre-exercise sphingolipid concentrations. In other words, the geometric means of the sphingolipid concentrations differ by a factor of 1.19 post- to pre-exercise in healthy individuals in the study conducted by Bergman et al. (1).

 Similarly, we used the data provided by Bergman et al. to calculate a standard deviation of log2-transformed sphingolipid levels of 0.407 and a correlation coefficient between pre-and post-intervention values of 0.8.

 We rewrote the section on sample size calculation as follows to make it clearer:

 “Due to the extensive number of targeted endpoints (n=61), a classical sample size calculation cannot be performed. Therefore, to estimate the required sample size, we based our analysis on the four most investigated sphingolipid species, which are also the species entering the ceramide scores used at the Mayo Clinic ( *i.e. *ceramide 16:0, ceramide 18:0, ceramide 24:0 and ceramide 24:1) (2, 3). Further, we hypothesised for simplicity that log2-transformed pre-intervention sphingolipid levels are similar in both the intervention and the control groups (due to the randomisation). Next, we assumed a standard deviation of log2-transformed sphingolipid levels of 0.407, an effect size (expressed as a geometric mean ratio) of 1.19 and a correlation coefficient between pre-and post-intervention values of 0.8. To obtain these values, we used the raw data of the four sphingolipids mentioned above issued from a previous exercise intervention study (1). Specifically, we calculated the geometric mean of the sphingolipid concentrations post- and pre-exercise for the four primary endpoints ( *i.e.* ceramide 16:0, ceramide 18:0, ceramide 24:0 and ceramide 24:1). We then calculated the ratio of the geometric means post- to pre-exercise. Finally, we averaged the geometric mean ratios obtained for these four endpoints and obtained a value of 1.194. We then log2-transformed 1.194 and obtained 1.19. Therefore, 1.19 is the log2-transformed geometric mean ratios of the post- to pre-exercise sphingolipid concentrations. In other words, the geometric means of the sphingolipid concentrations differ by a factor of 1.19 post- to pre-exercise in healthy individuals in the study conducted by Bergman et al. (1).”

**Statistical analysis: Since this is a randomized trial you should not include into the linear mixed effects model a main effect for the treatment group. The treatment group should be only included in the interaction term with timepoints itself. Randomization implies there is no group difference at baseline pre treatment, so both groups have the same intercept. Treatment can only causally act after the baseline timepoint.**

 Many thanks for pointing this out. We modified the model in consequence so that the two groups are assumed to be identical at the baseline measurements. The modified model allows for the groups to diverge solely at the time points following the intervention. Specifically, we omitted the fixed effects for treatment groups and included a three-way interaction term between the group, time point and an indicator variable for time points following the intervention.

 We rewrote the section on sample size calculation as follows to make it clearer:

 “The trajectory over time of each lipid species will be modelled using linear mixed models with a random effect for each subject. To identify variables, we should include in the models, we drew a causal-directed acyclic graph (DAG) using DAGitty. ^70^ Sex, body fat mass, CRF, and habitual physical activity were identified as variables to be included in the linear mixed models to reduce the outcome variation and improve the precision of the average causal effect of the intervention on the sphingolipidome (Figure 4). ^69^ As explained above, the design will control food intake as each participant will be provided with individualised, pre-packaged meals for days 7 to 10. It is also assumed that age difference will not play a significant role as all participants will be between 20 and 29 years old. Therefore, the models will include fixed effects for time points, sex, fat body mass, CRF, physical activity, and a three-way interaction term between the group, time point and an indicator variable for time points following the intervention. Planned contrasts between the groups at each time point following the intervention will allow assessing differences in lipid concentrations between the groups after the intervention.”

**Just as a minor remark, sphingolipids and ceramides are presented in the introduction only as having detrimental effects especially in the cell/molecular mechanism part. The literature is not clear so far to my knowledge e.g. how chain length of ceramides are related to CRF and CV risk. It seems that short chain ceramides have more "detrimental" effects while very long chain ceramides seem to have also "positive" effects. Saturation seems to play also a role: C24:0 is more positively associated with CRF and inversely with CVD mortality while C24:1 seems to be different. C16/C24 ratio is inversely associated with mortality and risk of CV incident events. See e.g. Peterson *et al.,* (2018) ^1^ . So maybe include 1-2 sentences into the introduction or discussion that the mechanistic picture is rather complex and it depends about which sphingolipid/ceramide in terms of chain-length and saturation we are speaking.**

 Thank you for this valuable comment. We added a sentence in the introduction, as kindly suggested, to better reflect the biological complexity of the sphingolipidome and its potential biological implications.

 These preliminary studies investigated, however, a limited number of sphingolipid species (n=8 and 7, respectively), whereas targeted lipidomics has now matured into a high-throughput approach allowing for comprehensive analysis of lipid metabolism at the molecular species level. ^5,6 ^This is particularly important as sphingolipids might have differential biological effects depending on acyl chain length and saturation, with shorter and unsaturated species being potentially more detrimental (4).

**After minor revision it should be accepted and I wish the authors good luck with conducting the study. I am highly looking forward to the upcoming and interesting results of this study.**

 Thank you for your insightful review.

 References

 1. Bergman BC, Brozinick JT, Strauss A, Bacon S, Kerege A, Bui HH, et al. Serum sphingolipids: relationships to insulin sensitivity and changes with exercise in humans. Am J Physiol Endocrinol Metab. 2015;309(4):E398-408.

 2. Nicholls M. Plasma ceramides and cardiac risk. European Heart Journal. 2017;38(18):1359-60.

 3. Vasile VC, Jaffe AS. An enhanced ceramide-based approach for primary prevention of atherosclerotic events. European Heart Journal Open. 2021;1(3).

 4. Peterson LR, Xanthakis V, Duncan MS, Gross S, Friedrich N, Völzke H, et al. Ceramide Remodeling and Risk of Cardiovascular Events and Mortality. Journal of the American Heart Association. 2018;7(10):e007931.
